# Effect of Dietary Patterns on Muscle Strength and Physical Performance in the Very Old: Findings from the Newcastle 85+ Study

**DOI:** 10.1371/journal.pone.0149699

**Published:** 2016-03-02

**Authors:** Antoneta Granic, Carol Jagger, Karen Davies, Ashley Adamson, Thomas Kirkwood, Tom R. Hill, Mario Siervo, John C. Mathers, Avan Aihie Sayer

**Affiliations:** 1 Newcastle University Institute for Ageing, Newcastle upon Tyne, United Kingdom; 2 Institute of Health & Society, Newcastle University, Newcastle upon Tyne, United Kingdom; 3 NIHR Newcastle Biomedical Research Centre in Ageing and Chronic Disease, and Newcastle upon Tyne Hospitals NHS Foundation Trust, Newcastle University, Newcastle upon Tyne, United Kingdom; 4 Ageing, Geriatrics & Epidemiology, Institute of Neuroscience, Newcastle University, Newcastle upon Tyne, United Kingdom; 5 Human Nutrition Research Centre, Newcastle University, Newcastle upon Tyne, United Kingdom; 6 Institute for Cell and Molecular Biosciences, Newcastle University, Newcastle upon Tyne, United Kingdom; 7 School of Agriculture, Food and Rural Development, Newcastle University, Newcastle upon Tyne, United Kingdom; 8 Institute of Cellular Medicine, Newcastle University, Newcastle upon Tyne, United Kingdom; 9 MRC Lifecourse Epidemiology Unit, University of Southampton, Southampton, United Kingdom; 10 NIHR Collaboration for Leadership in Applied Health Research and Care: Wessex, University of Southampton, Southampton, United Kingdom; University Of São Paulo, BRAZIL

## Abstract

**Background:**

Healthy diet has been associated with better muscle strength and physical performance in cross-sectional studies of older adults but the effect of dietary patterns (DP) on subsequent decline, particularly in the very old (aged 85+), has not been determined.

**Objective:**

We investigated the association between previously established DP and decline in muscle strength and physical performance in the very old.

**Design:**

791 participants (61.8% women) from the Newcastle 85+ Study were followed-up for change in hand grip strength (HGS) and Timed Up-and Go (TUG) test over 5 years (four waves 1.5 years apart). Mixed models were used to determine the effects of DP on muscle strength and physical performance in the entire cohort and separately by sex.

**Results:**

Previously we have established three DP that varied in intake of red meats, potato, gravy and butter and differed with key health and social factors. HGS declined linearly by 1.59 kg_F_ in men and 1.08 kg_*F*_ in women (both p<0.001), and TUG slowed by 0.13 log_10_-transformed seconds (log_10_-s) in men and 0.11 log_10_-s in women per wave after adjusting for important covariates (both p<0.001), and also showed a nonlinear change (p<0.001). Men in DP1 (‘High Red Meat’) had worse overall HGS (β = -1.70, p = 0.05), but men in DP3 (‘High Butter’) had a steeper decline (β = -0.63, p = 0.05) than men in DP2 (‘Low Meat’). Men in DP1 and women in DP3 also had overall slower TUG than those in DP2 (β = 0.08, p = 0.001 and β = 0.06, p = 0.01, respectively), but similar rate of decline after adjusting for sociodemographic, lifestyle, health, and functioning factors. The results for HGS and TUG were not affected by participants’ cognitive status.

**Conclusions:**

DP high in red meats, potato and gravy (DP1), or butter (DP3) may adversely affect muscle strength and physical performance in later life, independently of important covariates and cognitive status.

## Introduction

Muscle strength and physical performance are important indicators of the health status of older adults [1–4]. As average life expectancy continues to increase, many older adults will experience functional impairment especially in very old age (aged 85 and over) [5,6]. Progressive decline in muscle strength and physical performance quantified by objective measures such as hand grip strength (HGS) [7] and the Timed Up-and-Go test (TUG) [8] is associated with poor health outcomes, disability, cognitive decline, frailty, malnutrition, risk of falls, and mortality in adults aged 65 and over [1,2,4,9–16]. However less is known about the determinants of decline, particularly diet, in the very old (aged 85+) [17,18].

The majority of observational and nutritional intervention studies have investigated the impact of single macro- (e.g. protein) and micro-nutrients (e.g. vitamin D, B_12_, antioxidants) on skeletal muscle function but have yielded inconclusive results (reviewed in [19,20]) possibly because such strategies fail to account for the cumulative effects of (synergies between) the complex mixture of different foods within most diets. To understand the role of the whole diet on health and functioning outcomes, dietary pattern (DP) analysis became the methodology of choice. DP analysis utilises either *a priori* (or hypothesis-driven) or *a posteriori* (exploratory or data-driven) approach to derive patterns [21,22]. *A priori* developed DP are based on pre-defined dietary scores for a specific diet or dietary guidelines, in which higher scores indicate heathier DP and a higher intake of beneficial foods (e.g. whole grains, fruits and vegetables, and fish). This approach is limited because it does not necessarily capture the complexity of whole diet, and because it relies on the current state of knowledge of what represents a healthy diet. The *posteriori* approach uses factor or cluster analysis to derive DP and takes into account a total diet without prior hypotheses about the diet-health relationship. Cluster analysis has advantages over other procedures because it groups individuals into unique, non-overlapping groups based on similarities of food/nutrient consumption. Individuals within each cluster have a similar diet, which may be based on mean intake, frequency or absence/presence of particular food/nutrient [23].

Studies examining the effect of whole diet or DP on decline in muscle strength and physical performance in the very old are scarce. Utilising data from the Newcastle 85+ Study, we therefore aimed to determine the association between DP derived by cluster analysis and change in HGS and TUG over 5 years.

## Methods

### Subjects

The Newcastle 85+ Study, a prospective cohort study of over 1,000 participants born in 1921 has been described [24,25]. The study assessed a range of biological, psychological and social factors that affected health and functioning of very old adults (aged 85+) over 5 years, who lived in Newcastle and North Tyneside, UK. The analytic sample for the present study comprised of 791 participants (302 (38.2%) men, and 489 (61.8%) women) with assigned DP [26], multidimensional health assessment (including HGS and TUG) and general practice record review (GPrr). Participants were assessed at baseline (2006/07) and followed up at 1.5 (wave 2), 3 (wave 3), and 5 years (wave 4) at their place of residence (home or care facility) by trained research nurses. At baseline, 768 participants (97.1%) with assigned DP had a complete HGS measurement and 735 (92.9%) had TUG assessment.

### Ethics statement

The study was approved by the Newcastle & North Tyneside Local Research Ethics Committee 1. Signed consent was obtained from each participant or from their consultee (usually a relative) if they lacked the capacity to consent prior to study commencement.

### Measurements

#### Muscle strength

Hand grip strength (HGS) [7] was assessed using a hand-held dynamometer (Takei A5401 digital 0-100kg x 0.1kd LCD). In a standing position, with their arm hanging normally beside the body and an elbow angle of 180° approximately, participants were asked to squeeze the dynamometer as hard as possible to measure the maximal force for each hand. Two alternate measurements (in kg_F_) for each hand were recorded and the mean of four measurements for each participant (M, SD) was calculated, and used in the analysis.

#### Physical performance

Physical performance was assessed by the Timed Up-and-Go (TUG) test [8,27]. Participants were asked to get up from a chair (seat height 46 cm off the floor, with armrests) and walk as quickly and safely as possible up to and around a marker placed 3 m away, walk back to the chair, and sit down with their backs positioned against the back of the chair. The test was performed only once, and the use of walking aids (e.g. cane, walking frame, and wheeled walker) was recorded. The time needed to complete the task from the participant first attempting to rise from the chair to the moment when they sit back on the chair was recorded in seconds (s) with a stop watch.

#### Dietary assessment

Dietary assessment and validation of 24-hr multiple-pass dietary recall (24-hr MPR) in the Newcastle 85+ Study have been described [26,28]. A pilot study in a sub-sample of this cohort determined that 24-hr MPR delivers more accurate energy and nutrient estimates, and is more suited for individual dietary assessment of the very old (e.g. less burdensome and reliant on long-term memory) compared with the food frequency questionnaire (FFQ) [28]. Trained research nurses recorded a detailed intake of foods eaten on the previous day for each participant on two different days of the week (excluding Fridays and Saturdays) at least one week apart. Details of food intake included amount, type and brand, cooking method, time and meal occasion. Each food was allocated an unique food code (>2,000) and intakes (mean for 2 days) were entered in a Microsoft Access-based dietary data system, and further grouped into 118 food groups based on McCance and Widdowson’s composition-of-foods [26,29]. Each food group represented a 2-day average value (in grams). These were further combined into 33 food groups based on food/nutrient composition similarities, and classified as absent (coded 0) or present (coded 1) in the food intake of each participant. Of those food groups, 30 were used in the cluster analysis as described elsewhere [26] and in S1 Table.

#### Covariates

We included the following covariates in the multivariable analyses: (a) sociodemographic (sex, education (0–9 / 10–11 / ≥12 years); (b) lifestyle (smoking (never / current smoker / former smoker); physical activity (low (score 0–1) / moderate (score 2–6) / high (score 7–18))); (3) health-related factors (number of chronic diseases (continuous); body mass index (BMI) (continuous); season-specific serum vitamin D [30]); (c) diet-related factors (total energy (MJ) (continuous); diet change in past year (yes / no)) [26]; and function-related factors (dominant hand (right / left / ambidextrous), and use of walking aids at baseline and follow-ups (yes / no)). Self-reported physical activity was assessed with a purpose-designed physical activity questionnaire validated in a sub-group of this cohort. Participants were classified into three categories based on the physical activity scores derived from the frequency and intensity of physical activity performed per week, and this classification correlates highly with actigraphy [31].

### Statistical analysis

SPSS Two-Step clustering was used to derive DP as described previously [26]. Briefly, the best cluster solution was determined with 30 food groups by automatic selection and the Bayesian Information Criterion (BIC), and the robustness and stability of the three-DP solution was re-evaluated by random ordering of cases and by comparing DP characteristics [26].

#### Muscle strength and physical performance by DP

The differences between DP in raw HGS and TUG times were compared using ANOVA for normally distributed and Kruskal-Wallis test for non-normally distributed continuous variables (Table 1).

**Table 1:** Hand grip strength and Timed Up-and-Go raw scores by dietary patterns at baseline and follow-ups.

Physical performance/scores	All participants	DP1:‘High Red Meat’	DP2: ‘Low Meat’	DP3: ‘High Butter’	p*
	n = 791	n = 276	n = 260	n = 255	
**Hand grip strength and**					
**dominant hand**					
Baseline (n)	768	263	257	247	
kg_F_ (M, SD)	18.6 (7.9)	17.6 (7.6)	18.2 (7.8)	17.3 (7.6)	
Dominant hand % (n)					
right	92.0 (712)	91.7 (243)	93.8 (242)	90.4 (227)	
left	6.8 (53)	6.4 (17)	5.8 (15)	8.4 (21)	
ambidextrous	1.2 (9)	1.9 (5)	0.4 (1)	1.2 (3)	
Follow-up at 1.5 years (n)	594	198	208	188	
kg_F_ (M, SD)	17.0 (7.8)	17.0 (7.3)	17.3 (8.2)	16.6 (7.8)	
Follow-up at 3 years (n)	445	135	163	147	
kg_F_ (M, SD)	16.4 (7.3)	15.8 (7.0)	17.5 (7.5)	15.9 (7.1)	
Follow-up at 5 years (n)	291	86	116	89	
kg_F_ (M, SD)	14.9 (7.0)	15.1 (6.5)	15.7 (7.8)	13.7 (6.3)	
**Timed Up-and-Go (TUG) and**					
**use of walking aids**					
Baseline (n)	735	252	250	233	
s (M, SD)	18.6 (14.7)	19.5 (15.0)	16.6 (12.9)	19.9 (16.0)	<0.001
Use of walking aids % (n)					
yes	17.8 (131)	19.0 (48)	17.2 (43)	17.2 (40)	
Follow-up at 1.5 years (n)	541	179	196	166	
s (M, SD)	21.4 (17.1)	23.3 (21.7)	19.7 (11.9)	21.3 (16.6)	
Follow-up at 3 years (n)	396	116	152	128	
s (M, SD)	21.1 (17.2)	21.1 (15.4)	18.9 (10.2)	23.8 (23.8)	
Follow-up at 5 years (n)	271	85	108	78	
s (M, SD)	20.7 (12.0)	21.3 (11.6)	19.6 (12.8)	21.5 (11.2)	
Use of walking aids % (n)					
yes	26.1 (71)	24.7 (21)	25.9 (28)	27.8 (22)	

*One-way ANOVA (with Games-Howell *post hoc*) for normally distributed continuous variables, Kruskal-Wallis test for non-normally distributed continuous variables (untransformed), and Chi-square test for categorical variables. Only significant p values at α≤0.05 are reported.

#### Decline in muscle strength and physical performance by DP

HGS data (kg_F_) were normally distributed, whereas TUG times were positively skewed at each assessment (wave), and thus the latter were log_10_ transformed, and used as a continuous variable. Lower log_10_-s indicated quicker (better) TUG performance.

We utilised multilevel linear modeling [32] to examine: (a) the association between DP and HGS and TUG times at baseline, 1.5, 3 and 5-year follow-up; and (b) to identify baseline variables associated with the initial level and the rate of change in both HGS and TUG over the study period. DP2 was deemed to be the healthiest dietary pattern ([26]; S1 Table), and used as a referent in all analyses.

For HGS we fitted a series of linear growth curve models as follows: (a) with ‘time’ in study (coded as: baseline (0), 1.5 years (1), 3 years (2), and 5 years (3)) at within-person level (Level 1; to examine the linear trend of time), and DP at between-person levels (Level 2; to test whether intercept (initial status) varied by DP), and with an interaction of DP and time, to test for varying slopes (rate of change) by DP (Model 1); and (c) with additional adjustment for confounders associated with HGS and diet as identified in the literature (Model 2 (sociodemographic, health and functioning-related variables), and Model 3 (lifestyle variables)) (Table 2). Random effects terms included both intercept and slope of HGS over time.

**Table 2:** Parameter estimates^*^ (β coefficients) of growth curve models for hand grip strength (HGS) over 5 years by dietary patterns^†^.

Outcome	Effects/variable	Model 1		Model 2		Model 3	
		β (SE)	p	β (SE)	p	β (SE)	p
HGS (kg_F_)	Intercept						
Entire cohort	Time	-1.23 (0.11)	<0.001	-1.27 (0.12)	<0.001	-1.28 (0.12)	<0.001
	Dietary patterns						
	DP1 (‘High Red Meat’)	-0.67 (0.69)	0.33	-1.25 (0.47)	0.01	-0.92 (0.46)	0.05
	DP2 (‘Low Meat’) (ref)	0		0		0	
	DP3 (‘High Butter’)	-0.76 (0.70)	0.28	-0.63 (0.48)	0.19	-0.37 (0.46)	0.42
	Slopes						
	Dietary patterns X Time						
	DP1 X Time	0.01 (0.17)	0.94	0.01 (0.18)	0.98	0.01 (0.18)	0.97
	DP2 X Time (ref)	0		0		0	
	DP3 X Time	-0.11 (0.17)	0.53	-0.14 (0.18)	0.44	-0.15 (0.18)	0.90
HGS (kg_F_)	Intercept						
Men	Time	-1.56 (0.21)	<0.001	-1.59 (0.22)	<0.001	-1.59 (0.22)	<0.001
	Dietary patterns						
	DP1	-2.62 (0.92)	0.01	-1.99 (0.88)	0.03	-1.70 (0.86)	0.05
	DP2 (ref)	0		0		0	
	DP3	-1.17 (0.97)	0.23	-0.38 (0.93)	0.68	-0.08 (0.92)	0.93
	Slopes						
	Dietary patterns X Time						
	DP1 X Time	-0.16 (0.31)	0.61	-0.15 (0.32)	0.63	-0.16 (0.32)	0.61
	DP2 X Time (ref)	0		0		0	
	DP3 X Time	-0.57 (0.32)	0.08	-0.60 (0.32)	0.07	-0.63 (0.32)	0.05
HGS (kg_F_)	Intercept						
Women	Time	-1.03 (0.12)	<0.001	-1.06 (0.12)	<0.001	-1.08 (0.12)	<0.001
	Dietary patterns						
	DP1	-0.92 (0.50)	0.07	-0.53 (0.51)	0.30	-0.22 (0.50)	0.66
	DP2 (ref)	0		0		0	
	DP3	-0.81 (0.50)	0.10	-0.70 (0.50)	0.16	-0.50 (0.48)	0.29
	Slope						
	Dietary patterns X Time						
	DP1 X Time	0.22 (0.18)	0.22	0.11 (0.19)	0.55	0.13 (0.19)	0.49
	DP2 X Time	0		0		0	
	DP3 X Time	0.14 (0.17)	0.42	0.12 (0.18)	0.53	0.11 (0.18)	0.55

*Estimated β values (SE) of fixed effects using HGS longitudinal data. Random effects terms included both intercept and slopes of HGS scores over time. Time in the study was coded as baseline (0), 1.5-year follow-up (1), 3-year follow-up (2) and 5-year follow-up (3).

^†’^Low Meat’ (DP2) served as a referent group.

Model 1 includes a liner trend of time, dietary patterns, and their interaction term.

Model 2 is additionally adjusted for sex, education, dominant hand, diet change in the past year and health-related variables (season-specific serum vitamin D quartiles, total energy (MJ), number of chronic diseases, and BMI).

Model 3 is further adjusted for lifestyle variables (physical activity and smoking).

BMI, body mass index.

For TUG we examined both linear and quadratic effects of time (i.e. to account for nonlinear change over time) and their interaction terms over 5 years, and adjusted for a similar set of covariates including use of walking aids (Table 3). All predictors were time-invariant (baseline) except for walking aids use (Table 1). Random effects included both intercept and slope (linear) of TUG over time.

**Table 3:** Parameter estimates^*^ (β coefficients) of growth curve models for Timed up-and-go (TUG) test over 5 years by dietary patterns^†^.

Outcome	Effects/variable	Model 1		Model 2		Model 3	
		β (SE)	p	β (SE)	p	β (SE)	p
TUG (log_10_-s)	Intercept						
Entire cohort	Time	0.11 (0.01)	<0.001	0.11 (0.01)	<0.001	0.12 (0.01)	<0.001
	Time^2^	-0.02 (0.01)	<0.001	-0.03 (0.01)	<0.001	-0.03 (0.01)	<0.001
	Dietary patterns						
	DP1 (‘High Red Meat’)	0.06 (0.02)	0.003	0.05 (0.02)	0.01	0.03 (0.02)	0.052
	DP2 (‘Low Meat’) (ref)	0		0		0	
	DP3 (‘High Butter’)	0.06 (0.02)	0.01	0.06 (0.02)	<0.001	0.05 (0.02)	0.002
	Slopes						
	Dietary patterns X Time						
	DP1 X Time	-0.01 (0.02)	0.49	-0.02 (0.02)	0.25	-0.02 (0.02)	0.35
	DP2 X Time (ref)	0		0		0	
	DP3 X Time	-0.02 (0.02)	0.23	-0.05 (0.02)	0.02	-0.04 (0.02)	0.04
	Dietary patterns X Time^2^						
	DP1 X Time^2^	0.001 (0.01)	0.42	0.01 (0.01)	0.30	0.01 (0.01)	0.43
	DP2 X Time^2^	0		0		0	
	DP3 X Time^2^	0.10 (0.01)	0.15	0.01 (0.01)	0.04	0.01 (0.01)	0.06
TUG (log_10_-s)	Intercept						
Men	Time	0.13 (0.02)	<0.001	0.13 (0.02)	<0.001	0.13 (0.02)	<0.001
	Time^2^	-0.03 (0.01)	<0.001	-0.04 (0.01)	<0.001	-0.04 (0.01)	<0.001
	Dietary patterns						
	DP1	0.12 (0.03)	<0.001	0.09 (0.03)	0.001	0.08 (0.02)	0.001
	DP2 (ref)	0		0		0	
	DP3	0.08 (0.03)	0.02	0.05 (0.03)	0.09	0.04 (0.03)	0.11
	Slopes						
	Dietary patterns X Time						
	DP1 X Time	-0.04 (0.03)	0.17	-0.05 (0.03)	0.12	-0.04 (0.03)	0.12
	DP2 X Time (ref)	0		0		0	
	DP3 X Time	-0.04 (0.03)	0.18	-0.05 (0.03)	0.13	-0.04 (0.03)	0.17
	Dietary patterns X Time^2^						
	DP1 X Time^2^	0.02 (0.01)	0.1	0.02 (0.01)	0.11	0.02 (0.01)	0.13
	DP2 X Time^2^	0		0		0	
	DP3 X Time^2^	0.02 (0.01)	0.08	0.02 (0.01)	0.07	0.02 (0.01)	0.08
TUG (log_10_-s)	Intercept						
Women	Time	0.09 (0.02)	<0.001	0.11 (0.02)	<0.001	0.11 (0.02)	<0.001
	Time^2^	-0.02 (0.01)	0.001	-0.02 (0.01)	<0.001	-0.02 (0.01)	<0.001
	Dietary patterns						
	DP1	0.04 (0.03)	0.21	0.02 (0.02)	0.49	-0.01 (0.02)	0.64
	DP2 (ref)	0		0		0	
	DP3	0.05 (0.03)	0.06	0.07 (0.02)	0.002	0.06 (0.02)	0.01
	Slope						
	Dietary patterns X Time						
	DP1 X Time	6.8E-5 (0.03)	0.1	-0.01 (0.03)	0.86	0.003 (0.03)	0.91
	DP2 X Time	0		0		0	
	DP3 X Time	-0.01 (0.03)	0.62	-0.05 (0.03)	0.07	-0.04 (0.02)	0.1
	Dietary pattern X Time^2^						
	DP1 X Time^2^	-0.0001 (0.01)	0.99	0.001 (0.01)	0.88	-0.001 (0.01)	0.9
	DP2 X Time^2^	0		0		0	
	DP3 X Time^2^	0.01 (0.01)	0.59	0.01 (0.01)	0.2	0.01 (0.01)	0.27

*Estimated β values (SE) of fixed effects using TUG log_10_-transformed longitudinal data. Random effects terms included both intercept and slopes of TUG log_10_-transformed times over the study period. Time in the study was coded as baseline (0), 1.5-year follow-up (1), 3-year follow-up (2) and 5-year follow-up (3).

^†^’Low Meat’ (DP2) served as a referent group.

Model 1 includes a liner and quadratic trends of time, dietary patterns, and their interaction terms (DP X Time, DP X Time^2^).

Model 2 is additionally adjusted for sex, education, diet change in the past year, use of walking aids (time-varying covariate), and health-related factors (total energy (MJ), number of chronic diseases, BMI and season-specific vitamin D quartiles).

Model 3 is further adjusted for lifestyle factors (physical activity and smoking).

BMI, body mass index

The analyses for change in HGS and TUG by DP were also stratified by sex. Negative β estimates for HGS, and positive (increasing) β estimates for TUG indicated worse performance.

We used SPSS MIXED procedure (SPSS, 2002) with ‘REPEATED’ command, restricted maximum likelihood (RML), and diagonal and unstructured covariance matrix at Level 1 and Level 2, respectively to generate parameter estimates (β) for both outcomes.

All analyses were conducted using IBM SPSS (V.21; IBM Corporation, Armonk, NY, USA), and all statistics were 2-sided at α = 0.05.

### Sensitivity analysis

#### Characteristics of participants lost to follow-up

We compared participants lost to follow-up, through either withdrawal or death, with those still in the study 5 years post baseline by Mann-Whitney U test for ordered and non-normally distributed continuous, and by χ^2^ tests for categorical data.

#### Decline in muscle strength and physical performance by DP

For HGS we also examined nonlinear effect of time and added Time^2^ to the Model 1. All multilevel models were repeated for the entire cohort after excluding 59 participants diagnosed with dementia/Alzheimer’s disease (AD) (from GPrr) at baseline for both outcomes and in sex-stratified analyses. Mixed models for the TUG test were further adjusted for cognitive status at baseline (SMMSE scores ≥26 (normal) and SMMSE scores ≤25 (impaired)) [26,30].

Multicollinearity of confounders was assessed by examining the correlation matrix and multicollinearity diagnostics (i.e. Tolerance, Eigenvalues and Condition Index).

## Results

We examined the association between previously identified dietary patterns (DP1 (‘High Red Meat’), DP2 (‘Low Meat’), and DP3 (‘High Butter’)) [26] and change in muscle strength (HGS) and physical performance (TUG) across four measurement occasions (Table 1). Participants within each DP differed with respect to consumption of several food groups (including butter, unsaturated fats spreads and oils, gravy, potato/potato dishes and red meats/meat dishes), and to key sociodemographic and health measures (for details see [26]; S1 Table). Briefly, DP1 (‘High Red Meat’) had the highest proportion of participants consuming red meats/meat dishes, gravy, potato/potato dishes, and unsaturated fats spreads and oils, but the lowest proportion of participants eating butter compared with DP2 and DP3. DP2 (‘Low Meat’) had the lowest proportion of participants consuming meats (i.e. red meats/meat dishes, processed meats, and poultry), gravy and potato/potato dishes, and the highest proportion of those eating fruits, fish/seafood, whole grains/cereal product, dairy, and soups compared with others. DP3 (‘High Butter’) had the highest proportion of participants eating butter, and the lowest proportion of those consuming unsaturated fats spreads and oils compared with DP1 and DP2. Participants in DP2 were healthier (had less disability, depression and dementia, and better lipid profile), more physically active, and wealthier (more educated, belonged to higher social class, and lived in affluent areas), but were less likely to be married compared with others.

### Hand grip strength by DP over 5 years

The results of multilevel models investigating the association between DP and change in HGS over the study period are presented in Table 2. In the analysis with the entire cohort, HGS declined significantly over 5 years (p<0.001 in all models), and particularly in men (p<0.001). Specifically, HGS declined linearly by -1.28 kg_F_ per wave (1.5 years) in the entire cohort, and by -1.59 and -1.08 kg_F_ per wave in men and women, respectively after adjustment for covariates (DP, sex, education, dominant hand, diet change in past year, health-related factors (serum vitamin D, total energy (MJ), number of chronic diseases, BMI), and lifestyle factors (physical activity and smoking)) (Table 2, Model 3). A nonlinear (i.e. quadratic) effect of time was not significant (data not shown).

When tested as a main effect in the fully adjusted model (Model 3), DP1 (‘High Red Meat’) was associated with overall weaker HGS (β (SE) = -0.92 (0.46), p = 0.05) compared with DP2 (‘Low Meat’) in both the entire cohort, and in men (-1.70 (0.86), p = 0.05). However, the rates of decline (slopes) in HGS did not differ by DP over 5 years, although men belonging to DP3 (‘High Butter’) experienced a steeper loss in grip strength (-0.63 (0.32), p = 0.05) compared with those in DP2 (Model 3, Table 2). No association between DP and HGS was observed in women (S1 Fig).

### Timed Up-and-Go Test by DP over 5 years

There was a significant linear decline in TUG (log_10_-transformed) means (log_10_-s) over four waves, indicating slower or poorer performance in the entire cohort, men and women (p<0.001 in all) (Model 1 to 3, Table 3). Specifically, the overall average TUG speed declined by 0.12 log_10_-s per wave after adjustment for potential covariates, and by 0.13 and 0.11 in men and women, respectively (Model 3, Table 3). There was a significant quadratic effect of time in the entire cohort (β (SE) for all participants = -0.03 (0.004)), men (-0.04 (0.01)), and women (-0.02 (0.01)) (p<0.001 for all) (Model 3), suggesting a nonlinear rate of change in TUG over time (i.e. the rate of change decelerated over the 5 years) (S2 Fig).

In the fully adjusted model (Table 3, Model 3), DP3 (‘High Butter’) was associated with overall longer TUG times (0.05 (0.02), p = 0.002), and faster rate of linear decline (i.e. more time taken to complete the task over the study period) (-0.04 (0.02), p = 0.04) compared with those in DP2. In sex-stratified analysis, the association was significant for men belonging to DP1 (‘High Red Meat’) (0.08 (0.02), p = 0.001), and for women in DP3 (0.06 (0.02), p = 0.01), indicating worse overall TUG performance compared with DP2. Slopes (rates of decline) did not differ across DP for either linear or quadratic terms for both men and women, indicating no effect of DP on TUG test change or on change in rate of change over time.

### Results for sensitivity analyses

#### Characteristics of participants lost to follow-up 5 years post baseline

Compared with participants with assigned DP and HGS data 5 years later (n = 291), those lost to follow-up (n = 477 (62.1%)) were more likely to be women (p = 0.04), to be cognitively impaired (p = 0.001) and depressed (p = 0.02), and less physically active (p = 0.02) at baseline. Those who stayed in the study were more likely to belong to middle quartiles of sex-specific vitamin D (p = 0.02). DP was not associated with loss to follow-up (data not shown).

Similarly, compared with participants with assigned DP and TUG data 5 years later (n = 271), those lost to follow-up (n = 464 (63.1%)) were more likely to be cognitively impaired (p = 0.001), depressed (p = 0.02), and to be less physically active (p = 0.02) at baseline. Again, DP was not associated with loss to follow-up (data not shown).

#### Decline in muscle strength and physical performance by DP

Repeating the multilevel modeling for HGS after excluding 59 participants diagnosed with dementia/AD; the results were unchanged (data not shown). Also, adjusting the final model (Model 3) for cognitive status at baseline to account for any cognitive component of the TUG test did not alter the conclusions (data not shown).

## Discussion

In the present study we investigated the relationship between previously defined DP [26] and muscle strength and physical performance (characterised by HGS and TUG, respectively) in very old participants from the Newcastle 85+ Study. To the best of our knowledge, this is the first such prospective evaluation of the impact of DP on HGS and TUG in this age group. DP1 (‘High Red Meat’), a dietary pattern high (i.e. the highest proportion of people) in red meats/meat dishes, gravy and potato [26] was associated with overall worse HGS in the entire cohort and in men, but men in DP3 (‘High Butter’), a dietary pattern high in butter and low in unsaturated fats had a steeper rate of decline in HGS (by -0.63 kg_*F*_ per wave) over 5 years compared with DP2 (‘Low Meat’), a dietary pattern low (i.e. the lowest proportion of people) in red/processed meats, gravy and potato. In the entire cohort, participants in DP3 had overall poorer TUG performance and required longer time to complete the TUG over 5 years compared with those in DP2 after adjustment for important covariates. Men in DP1 and women in DP3 had worse overall TUG times, but the rate of decline did not differ by DP. Excluding participants diagnosed with dementia/AD at baseline or adjusting for cognitive status (i.e. SMMSE) did not change the results, which infers that the age-related decline which we observed is not due to cognitive impairment. Participants in DP2 had reached very old age more physically fit compared with their peers in the other DPs, irrespective of their cognitive health.

Existing studies of physical performance and muscle function in older adults (aged 60+) have investigated a limited number of nutrients (reviewed in [19,20]), such as protein [20], vitamin D (serum and dietary) (e.g. [33,34]), and magnesium [35]. However, despite the recognised utility of a single nutrient approach for testing muscle heath-diet hypotheses [36], focusing on consumption of a single nutrient or food group may be of limited value from a public health perspective because it ignores the likely cumulative, synergistic/antagonistic effect of foods/nutrients within complex diets on muscle strength and physical performance. Some observational studies have investigated the cross-sectional associations between DP (derived *a posteriori* using Principal Component Analysis), muscle strength and physical performance in older adults (e.g. [37–39]) but none in the very old. For example, in the Hertfordshire Cohort Study of ~3000 older men and women (aged 59–73), a higher ‘prudent’ dietary score (characterised by high intake of fruits, vegetables, fatty fish and breakfast cereal) was associated with stronger hand grip [37]. Additional analysis of those aged 63–73 from this cohort [38] revealed a faster 3-m walk times in women consuming diets with the higher ‘prudent’ score. Similarly, we found that our healthiest dietary pattern (DP2) which was high in fruits, fish/sea food, eggs, nuts and whole grains but low in red/processed meats and potato/potato dishes (DP2) [26] was associated with stronger hand grip and faster TUG performance compared with those with the less healthy dietary patterns characterised by a higher intake of red meats (including processed meats, ham and bacon) and butter independently of several important covariates (S1 and S2 Figs). The benefits of DP2 on muscle strength/performance were independent of the effects of other health-related behaviours (physical activity, smoking, BMI), disease burden, and vitamin D and cognitive status—factors reported to be associated with grip strength and physical performance decline in older adults [2,34,35,40]. Furthermore, in the 1999–2002 National Health and Nutrition Examination Survey, higher overall diet quality (determined by the Healthy Eating Index-2005 using *a priori* approach) was associated with faster gait speed and stronger knee extensor power in a nationally representative sample of U.S. older adults aged 60 and over [39].

There have been some longitudinal studies in young old adults linking DP to subsequent decline in physical performance [41,42]. In the InCHIANTI study, higher adherence to Mediterranean-style diet was associated with less decline in lower extremity function (mobility) over 9 years in participants aged 65+ [41], and with slower decline over 8 years in usual and rapid 20-m walking speed among 2000 older adults aged 70+ in the Health, Aging, and Body Composition study [42].

Several biological mechanisms may have played a role in the poorer physical performance and muscle strength in participants belonging to DP1 (‘High Red Meat’) and DP3 (‘High Butter’). At a cellular level, older adults experience a progressive loss of lean muscle tissue, especially in very old adulthood [43], a concomitant increase in intramyocellular lipids [44], diminished muscle protein synthesis from the available amino acids pool (i.e. ‘anabolic resistance’) [45], and declining oxidative capacity, quality, and number of mitochondria [46]. Participants in DP3 (‘High Butter’), the DP highest in total fat (and percent energy (%E) from fat), cholesterol, saturated fatty acids (SFA) (and %E from SFA), and monounsaturated fatty acids (MUFA), but lowest in polyunsaturated fatty acids (PUFA) and MUFA/SFA ratio and %E from carbohydrates [26] may have compromised aged muscle fibers through potentially increased lipid deposition, insulin resistance, and inflammation, and thus diminished overall muscle quality and composition [47]. Indeed, experimental studies in older rodents have shown that a high-fat diet induces insulin resistance and a strong increase in ectopic fat deposition in muscle cells, and reduced muscle mitochondrial activity and muscle protein anabolism [48,49]. In contrast, animals fed a diet high in ω-6 PUFA showed improved insulin sensitivity and reduced intramyocellular lipid accumulation compared to those consuming a high-SFA diet [50,51].

Several studies have suggested a role for muscle quality in functional decline in older adults as possible explanation for the apparent disconnect between age-related changes in muscle mass and in muscle strength—the latter being more profound with ageing [47]. Although we did not observe statistically significant differences in fat mass across DP (i.e. total body adiposity estimated by bioelectrical impedance), participants in DP3 had a lower lean muscle mass compared with those in DP1 and DP2 (p = 0.003; details not shown). Thus, a combination of lower lean mass and poorer muscle quality in participants consuming DP3 may have contributed to a faster rate of decline in TUG and a steeper loss of HGS in men over 5 years compared with those in DP2 (‘Low Meat’). Similarly, despite having higher lean muscle mass, the quality of muscle may have been compromised in participants belonging to DP1.

Of course, moderate consumption of lean red meat (an excellent source of protein, leucine, creatine, vitamins (e.g. B12) and minerals (iron and zinc)) may be beneficial in maintenance of muscle mass in older adults [52,53]. Indeed, a recent randomized control trial of 100 women aged 70–90 has shown that consumption of cooked lean red meat (~160 g/day 6 days a week for 4 months) in combination with resistance training improved total lean muscle mass and function [54]. Also, leg lean muscle mass and leg extension increased in older women (aged 65–70) consuming a healthy diet (characterised by ω-6/ω-3 PUFA < 2 ratio; MUFA and PUFA from plant oils as main source of fat; nuts and seeds, and 20%E of protein from lean meat, low fat dairy, and fish/sea food) for 6 months in combination with resistant training [55]. The latter diet has similarities with DP2 (‘Low Meat’), which had the highest percentage of participants consuming potentially more healthy foods for muscle quality, mass and strength (fish/sea food, dairy, nuts, whole grains/cereal, fruits) and overall health ([26]; S1 Table).

Another possible mechanism may be related to the effects of an unfavourable acid-base balance for muscle health in DP1, as a greater dietary acidity has been implicated in muscle loss and decline in physical performance [56,57]. Based on estimated net endogenous acid production (calculated using the Frassetto et al [58] algorithm), DP2 (‘Low Meat’), the DP high in alkalizing fruits and vegetables and low in acid-producing meats, had a lower acidity compared with DP1 (p = 0.01; details not shown), which may have resulted in more balanced diet for maintenance of lean muscle mass. Additionally, although we adjusted for disease burden (multi-morbidity), the presence of greater vascular pathology among participants in DP1 [26] may have contributed to reduced physical activity and, therefore muscle function, through vascular pathways. Participants following DP2 had better overall, including cardiovascular, health [26], which may have contributed to their better physical functioning.

Finally, it is important not to assume that the label (title) given to a DP represents the only, or even the most biologically, important aspect/component of that DP. All DP are complex and contain constellations of foods and nutrients with multiple effects on metabolism and health. When interpreting the potential causal relationships between DP and functional outcomes, it is also important to consider the foods/nutrients which are missing from (or present in low amounts in) a given DP as those which characterise a particular dietary pattern. In this case, the lower consumption of fruits, nuts, fish and sea food, eggs, soups and dairy among DP1 participants may be mechanistically important.

Taken together, these and the findings from our study indicate that a healthy dietary pattern characterised by higher consumption of fish, fruits, vegetables, nuts, cereal, dairy and oils rich in unsaturated fatty acids may ameliorate age-related functional decline and associated adverse health outcomes [2–4] independently of other health-related factors. If replicated in other populations, these findings may be an important foundation for public health policies and interventions to facilitate healthy ageing. In particular, we have shown that diets high in red/processed meats, gravy or butter but low in beneficial foods (e.g. fish and plant-based foods) may have negative effects on muscle strength and physical performance, and exacerbate age-related decline [59] at very old age especially in men. Conversely adherence to a healthy dietary pattern may have a long-lasting effect on physical function well into late adulthood, irrespective of cognitive status, disease burden and physical activity.

### Study strengths and limitations

The findings reported here should be interpreted with caution for the following reasons. First, several residual or unknown confounders may have influenced the final DP solution. Diet was evaluated only once (at baseline) by 24-hr MPR on two non-consecutive days excluding Friday’s and Saturday’s and spanning over four seasons, and, at this life stage, it is not known whether dietary patterns remain stable over 5 years [26]. In addition, seasonal variation in food consumption and differential eating patterns at weekends compared with weekdays may have contributed to less robust measures of habitual diet for specific individuals. Although physical performance decline was independent of cognitive status in this cohort, dietary choices may have been affected by predementia states, and may have contributed to dietary misreporting. To account for recent changes in diet, we included the variable ‘diet change in previous year’ in models but the reasons for the change and motivations for healthy food choices in older adults [60] were not explored. Other diet-related factors such as oral health, appetite loss, every day emotions, social support, food accessibility, dietary knowledge [61] may have influenced food choices. Furthermore, although we used several analytic techniques to confirm the robustness of DP [26], cluster analysis is exploratory and the food groupings that were employed may have affected the final DP solution.

Secondly, other factors may still explain the DP-physical function relationship. We have shown previously that higher education was an important predictor of potentially healthier diet, in addition to other socioeconomic indicators [26]. Participants in DP2 (‘Low Meat’) were healthier and more physically active compared with others, and may have had more interest, motivation, resources and knowledge to sustain healthier lifestyles (including diet) over longer periods of time prior to the study. The rate of decline in HGS (except for men in DP3) did not differ by DP, possibly due to loss of power over the 5 years of follow-up, with later values being influenced by selection bias through inclusion very robust older ‘survivors’ [5]. About half of the cohort were lost by the end of the study [62], and those who remained were healthier and more likely to belong to DP2 (‘Low Meat’) at baseline. β coefficients (log_10_-transformed seconds) for TUG were small, and may have limited interpretability and clinical relevance. Finally, the observed association between DP1 (‘High Red Meat’) and DP3 (‘High Butter’) and poorer physical performance may be confounded by unknown and uncontrolled factors affecting muscle strength and function (e.g. medication dosage, duration, and interaction, and motivation to perform the test).

Our study has several strengths, including its prospective design and use of objective measures of muscle strength and physical performance, representativeness of UK population (living in community and care facilities), validated dietary assessment [28], robustness of clustering technique used to derive DP [26], and adjustment for several known factors which influence muscle strength and function including serum vitamin D [30].

In conclusion, we have observed associations between *a posteriori* derived dietary patterns DP1 (a ‘High Red Meat’) and DP3 (‘High Butter’) and worse overall HGS and TUG independent of key risk factors for loss of muscle strength and decline in physical performance. These effects were more pronounced in men and were not affected by cognitive status. The results need to be confirmed in other prospective observational as well as intervention studies to identify the optimal dietary patterns for maintenance of physical function in very late adulthood.

## Supporting Information

S1 Fig Similar rate of decline in HGS across DP except in men. Compared with DP2 (‘Low Meat’) (black line with squares), men in DP1 (‘High Red Meat’) (grey line with diamonds) has worse overall HGS, but men in DP3 (‘High Butter’) (black line with triangles) had a steeper rate of decline over 5 years (B) after adjustment for socioeconomic factors (sex and education), dominant hand, dietary change in past year, health-related (season-specific serum vitamin D, total energy, number of chronic diseases, BMI), and lifestyle factors (physical activity and smoking). The rate of change in HGS did not vary by DP in the entire cohort (A) and in women (C). Time was coded as 0 (baseline), 1 (1.5-year follow-up), 2 (3-year follow-up) and 3 (5-year follow-up). Additional time points coded 4 and 5 were added to estimate trajectories in HGS by DP.
(TIF)

S2 Fig Change in TUG across DP. The growth curves represent β estimates of the fully adjusted model (Model 3). Greater log_10_-s indicated worse (slower) TUG performance. Participants in DP3 (‘High Butter’) (black line with triangles) had overall slower TUG performance and needed more time to complete the task over the study period compared with those in DP2 (‘Low Meat’) (black line with squares) (A). Men in DP1 (‘High Red Meat’) (grey line with diamonds) and women in DP3 (‘High Butter’) (black line with triangles) had worse overall TUG times compared with those in DP2 (‘Low Meat’) (black line with squares), but declined similarly over 5 year (B and C, respectively). Time was coded as 0 (baseline), 1 (1.5-year follow-up), 2 (3-year follow-up) and 3 (5-year follow-up). Additional time points coded 4 and 5 were added to estimate trajectories in TUG by DP.
(TIF)

S1 Table Main characteristics of dietary patterns in the Newcastle 85+ Study. (DOCX)
