# Supplementary figures and images for: Effect of Dietary Patterns on Muscle Strength and Physical Performance in the Very Old: Findings from the Newcastle 85+ Study
Source: PLoS One. 2016 Mar 2;11(3):e0149699. doi: 10.1371/journal.pone.0149699 (PMC4774908; doi:10.1371/journal.pone.0149699)

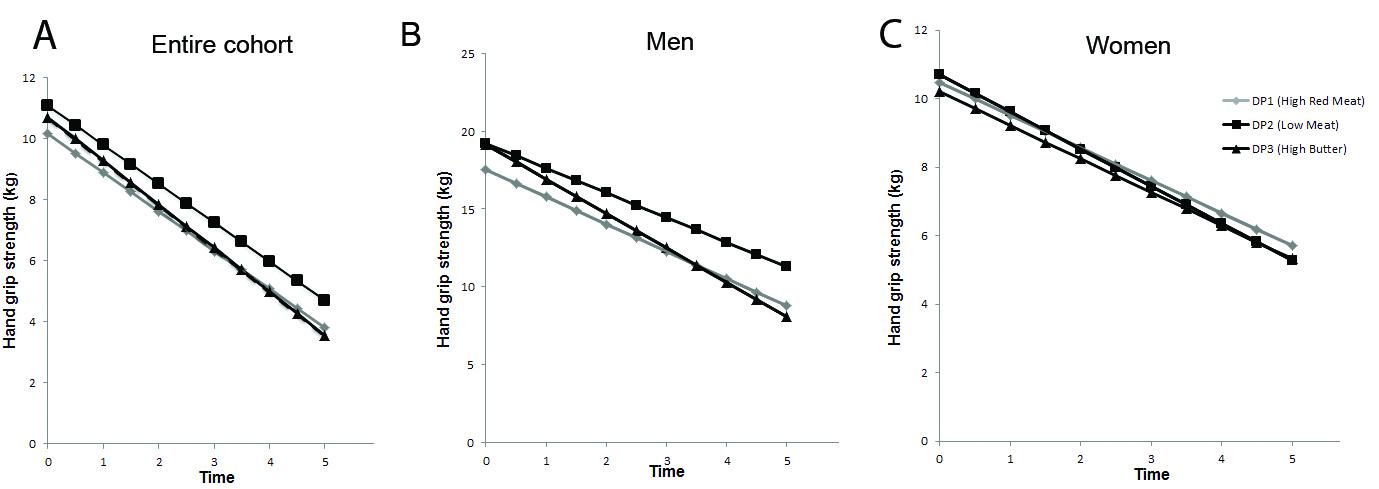

Supplement: S1 Fig — Compared with DP2 (‘Low Meat’) (black line with squares), men in DP1 (‘High Red Meat’) (grey line with diamonds) has worse overall HGS, but men in DP3 (‘High Butter’) (black line with triangles) had a steeper rate of decline over 5 years (B) after adjustment for socioeconomic factors (sex and education), dominant hand, dietary change in past year, health-related (season-specific serum vitamin D, total energy, number of chronic diseases, BMI), and lifestyle factors (physical activity and smoking). The rate of change in HGS did not vary by DP in the entire cohort (A) and in women (C). Time was coded as 0 (baseline), 1 (1.5-year follow-up), 2 (3-year follow-up) and 3 (5-year follow-up). Additional time points coded 4 and 5 were added to estimate trajectories in HGS by DP. (TIF) [file pone.0149699.s001.tif]

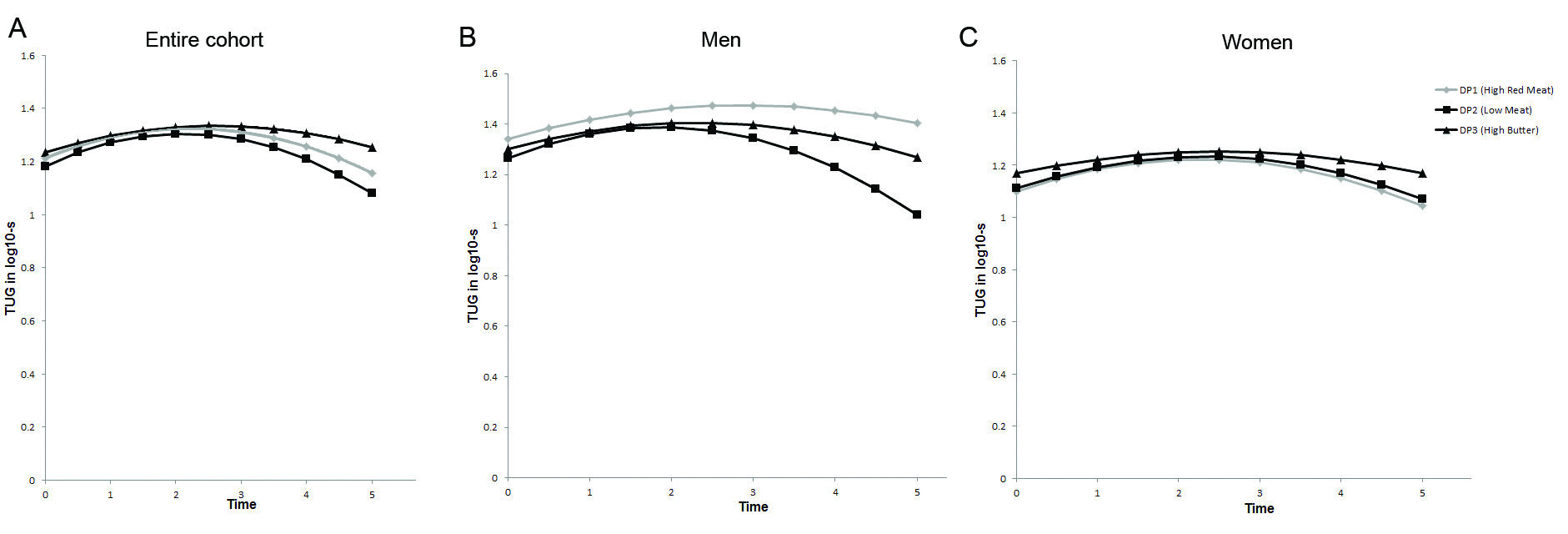

Supplement: S2 Fig — The growth curves represent β estimates of the fully adjusted model (Model 3). Greater log10-s indicated worse (slower) TUG performance. Participants in DP3 (‘High Butter’) (black line with triangles) had overall slower TUG performance and needed more time to complete the task over the study period compared with those in DP2 (‘Low Meat’) (black line with squares) (A). Men in DP1 (‘High Red Meat’) (grey line with diamonds) and women in DP3 (‘High Butter’) (black line with triangles) had worse overall TUG times compared with those in DP2 (‘Low Meat’) (black line with squares), but declined similarly over 5 year (B and C, respectively). Time was coded as 0 (baseline), 1 (1.5-year follow-up), 2 (3-year follow-up) and 3 (5-year follow-up). Additional time points coded 4 and 5 were added to estimate trajectories in TUG by DP. (TIF) [file pone.0149699.s002.tif]
