# Supplementary material for: Effect of Dietary Patterns on Muscle Strength and Physical Performance in the Very Old: Findings from the Newcastle 85+ Study
Source: PLoS One. 2016 Mar 2;11(3):e0149699. doi: 10.1371/journal.pone.0149699 (PMC4774908; doi:10.1371/journal.pone.0149699)
Supplement: S1 Table — (DOCX) [file pone.0149699.s003.docx]

**S1Table**. Main characteristics of dietary patterns* in the Newcastle 85+ Study

| Characteristics | DP1 (‘High Red Meat’) | DP2 (‘Low Meat’) | DP3 (‘High Butter’) |
| --- | --- | --- | --- |
| Main food groups’ differences | **Highest** (i.e. the highest proportion of | **Lowest** in meats/meat dishes (red and | **Highest** in butter and the **highest** g/day |
|  | people) in red meats/meat dishes, | processed meats, bacon and ham, and | of intake in butter. **Moderate** in red |
|  | potatoes/potato dishes, gravy, legumes | poultry), gravy and potatoes/potato | meats/meat dishes. **Low** in unsaturated |
|  | (including baked beans), and unsaturated | dishes, and **lowest** mean g/day of intake | and saturated fats spreads and oils. |
|  | fats spreads, and the **highest** mean g/day | of these foods. **Moderate** in butter. |  |
|  | intake of these foods. **Lowest** in butter | **Highest** **proportion** of people reported |  |
|  | (percentage and g/day). | eating fruits, nuts, whole grains and |  |
|  |  | cereal products, fish and sea food, eggs, |  |
|  |  | soups, low and high fat dairy, coffee and |  |
|  |  | alcohol, and the **highest** mean g/day of |  |
|  |  | intake of these foods. Regarded as the |  |
|  |  | healthiest DP and used as referent. |  |
| Nutrition-related values | **Highest** percent energy (%E) from | **Lowest** in protein. **Lowest** total plasma | **Highest** in total fat, cholesterol, MUFA, |
|  | starch and protein. | cholesterol and the **highest** HDL- | and SFA. **Lowest** in PUFA and |
|  |  | cholesterol concentration. | MUFA/SFA ratio. **Highest** percent |
|  |  |  | energy (%E) from fat and SFA. **Highest** |
|  |  |  | food energy density. **Lowest** in %E from |
|  |  |  | carbohydrates. |
| Sociodemographic factors | **More** likely to live in institutions. | **More advantaged** compared to others |  |
|  |  | (higher education and social class [higher |  |
|  |  | managerial/administrative occupations], |  |
|  |  | more likely to own their homes, and to |  |
|  |  | live in affluent areas). **More** likely to be |  |
|  |  | single. |  |
| Health-related factors | **More likely** to be non-smokers, but to be | **Healthier** compared to others (the least |  |
|  | diagnosed with cardiovascular disease. | likely to be disabled, depressed, obese, |  |
|  | **Higher** fat-free mass. | cognitively impaired or diagnosed with |  |
|  |  | dementia at baseline). **More** physically |  |
|  |  | active. **More** likely to take supplements. |  |
|  |  | **More** likely to drink alcohol. **More** |  |
|  |  | likely to be APOE ɛ4 negative. |  |

*The following 30 food groups were used in cluster analysis: (1) **butter**; (2) **unsaturated fat spreads and oils**; (3) **gravy**; (4) **potato and potato dishes**; (5) **red meats and meat dishes**; (6) **legumes**; (7) **coffee**; (8) **snacks and savouries**; (9) **vegetable**; (10) **low fat dairy**; (11) fruits; (12) nuts; (13) refrained grains and cereal products; (14) whole grains and cereal products; (15) fish and sea food; (16) bacon and ham; (17) poultry; (18) processed and other meats; (19) eggs; (20) soups; (21) saturated fat spreads and margarine; (22) high fat dairy; (23) preserves and syrups; (24) chocolate; (25) biscuits and cakes; (26) desserts and sweets; (27) tea; (28) alcohol; (29) hot drinks, and (30) soft sugary drinks. First 10 foods (in bold) contributed the most to DP separation, and are listed in descending order by the importance factor score [26].

APOE, apolipoprotein E; HDL, high density lipoprotein; MUFA, monounsaturated fatty acids; PUFA, polyunsaturated fatty acids; SFA, saturated fatty acids.
